# The genome sequence of a soldier beetle, *Cantharis flavilabris *Fallén, 1807

**DOI:** 10.12688/wellcomeopenres.22422.1

**Published:** 2024-06-11

**Authors:** Maxwell V. L. Barclay, Natural History Museum Genome Acquisition Lab, Darwin Tree of Life Barcoding collective, Wellcome Sanger Institute Tree of Life Management, Samples and Laboratory team, Wellcome Sanger Institute Scientific Operations: Sequencing Operations, Wellcome Sanger Institute Tree of Life Core Informatics team, Tree of Life Core Informatics collective, Darwin Tree of Life Consortium

**Affiliations:** 1 Natural History Museum, London, England, UK

**Keywords:** Cantharis flavilabris, soldier beetle, genome sequence, chromosomal, Coleoptera

## Abstract

We present a genome assembly from an individual female *Cantharis flavilabris* (soldier beetle; Arthropoda; Insecta; Coleoptera; Cantharidae). The genome sequence is 348.3 megabases in span. Most of the assembly is scaffolded into 7 chromosomal pseudomolecules, including the X sex chromosome. The mitochondrial genome has also been assembled and is 17.5 kilobases in length. Gene annotation of this assembly on Ensembl identified 22,711 protein coding genes.

## Species taxonomy

Eukaryota; Opisthokonta; Metazoa; Eumetazoa; Bilateria; Protostomia; Ecdysozoa; Panarthropoda; Arthropoda; Mandibulata; Pancrustacea; Hexapoda; Insecta; Dicondylia; Pterygota; Neoptera; Endopterygota; Coleoptera; Polyphaga; Elateriformia; Elateroidea; Cantharidae; Cantharinae; *Cantharis*; *Cantharis flavilabris* Fallén, 1807 (NCBI:txid877752).

## Background

The genome of the soldier beetle, *Cantharis flavilabris*, was sequenced as part of the Darwin Tree of Life Project, a collaborative effort to sequence all named eukaryotic species in the Atlantic Archipelago of Britain and Ireland. Here we present a chromosomally complete genome sequence for *Cantharis flavilabris*, based on one female specimen from Leith Hill, England, UK.

## Genome sequence report

**Figure 1.**
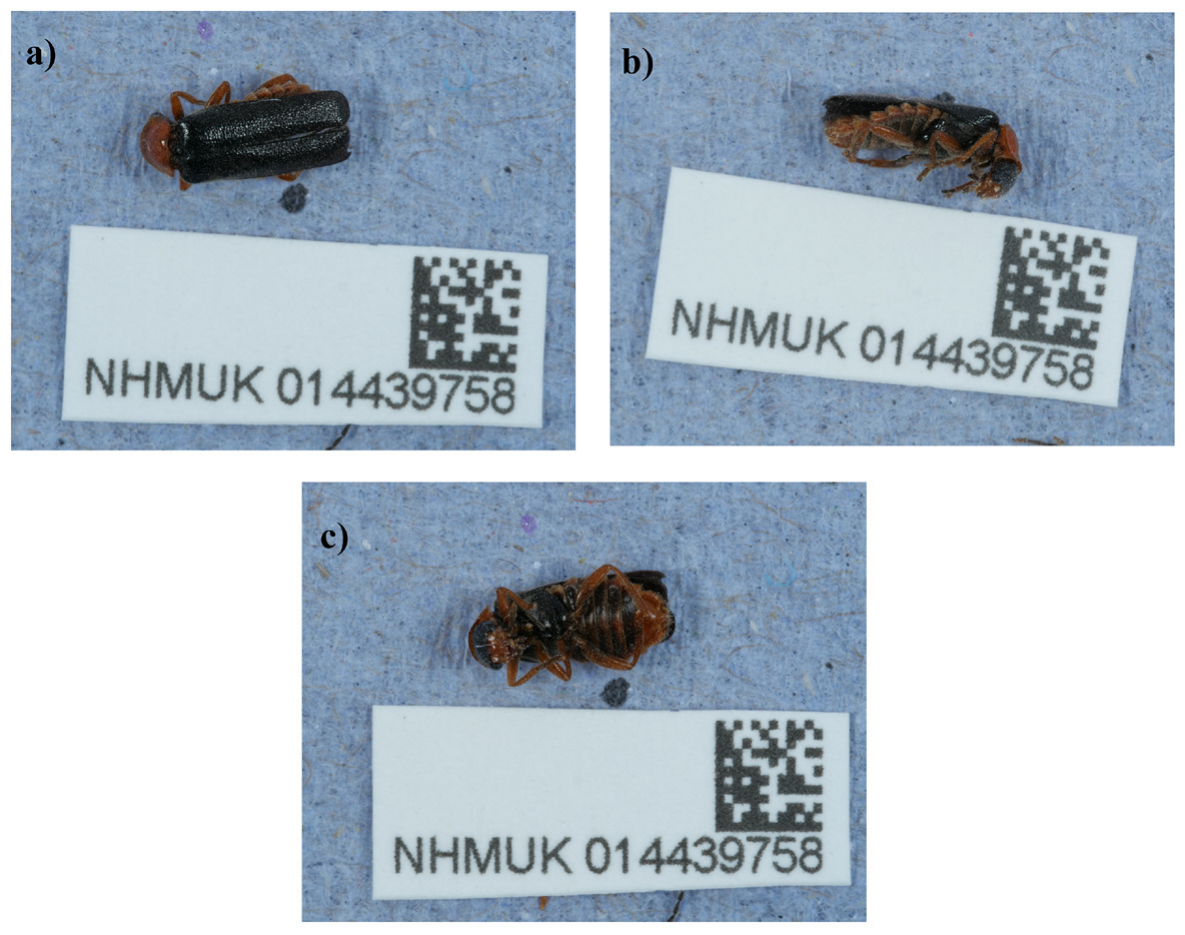
Photograph of the *Cantharis flavilabris* (icCanFlav1) specimen used for genome sequencing.

The genome was sequenced from one female *Cantharis flavilabris* ( Figure 1) collected from Leith Hill, England, UK (51.18, –0.37). A total of 74-fold coverage in Pacific Biosciences single-molecule HiFi long reads was generated. Primary assembly contigs were scaffolded with chromosome conformation Hi-C data. Manual assembly curation corrected 16 missing joins or mis-joins and removed 5 haplotypic duplications, reducing the assembly length by 0.60% and the scaffold number by 6.90%, and increasing the scaffold N50 by 11.36%.

**Figure 2.**
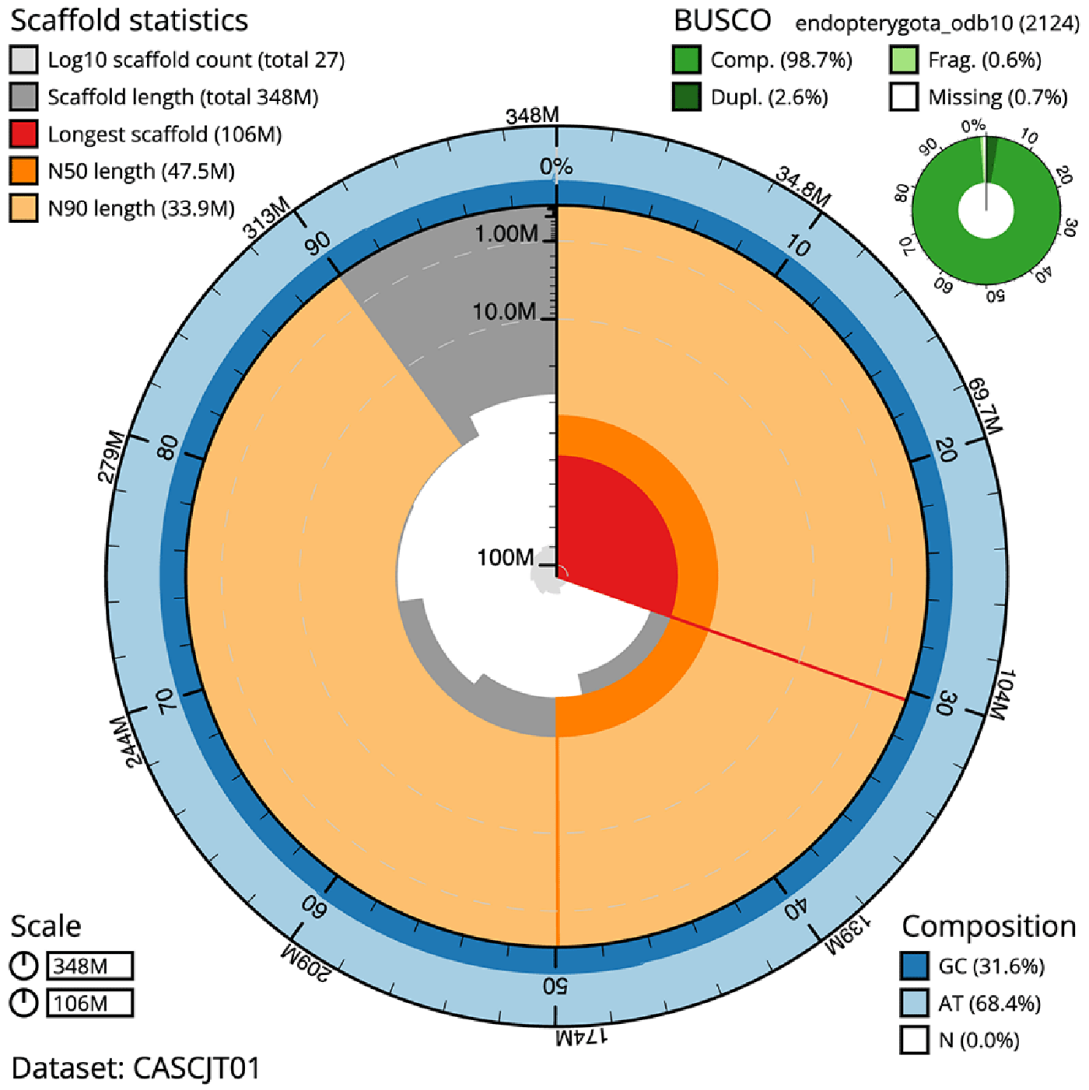
Genome assembly of *Cantharis flavilabris*, icCanFlav1.1: metrics. The BlobToolKit Snailplot shows N50 metrics and BUSCO gene completeness. The main plot is divided into 1,000 size-ordered bins around the circumference with each bin representing 0.1% of the 348,304,867 bp assembly. The distribution of scaffold lengths is shown in dark grey with the plot radius scaled to the longest scaffold present in the assembly (106,117,081 bp, shown in red). Orange and pale-orange arcs show the N50 and N90 scaffold lengths (47,548,398 and 33,898,588 bp), respectively. The pale grey spiral shows the cumulative scaffold count on a log scale with white scale lines showing successive orders of magnitude. The blue and pale-blue area around the outside of the plot shows the distribution of GC, AT and N percentages in the same bins as the inner plot. A summary of complete, fragmented, duplicated and missing BUSCO genes in the endopterygota_odb10 set is shown in the top right. An interactive version of this figure is available at https://blobtoolkit.genomehubs.org/view/CASCJT01/dataset/CASCJT01/snail.

**Figure 3.**
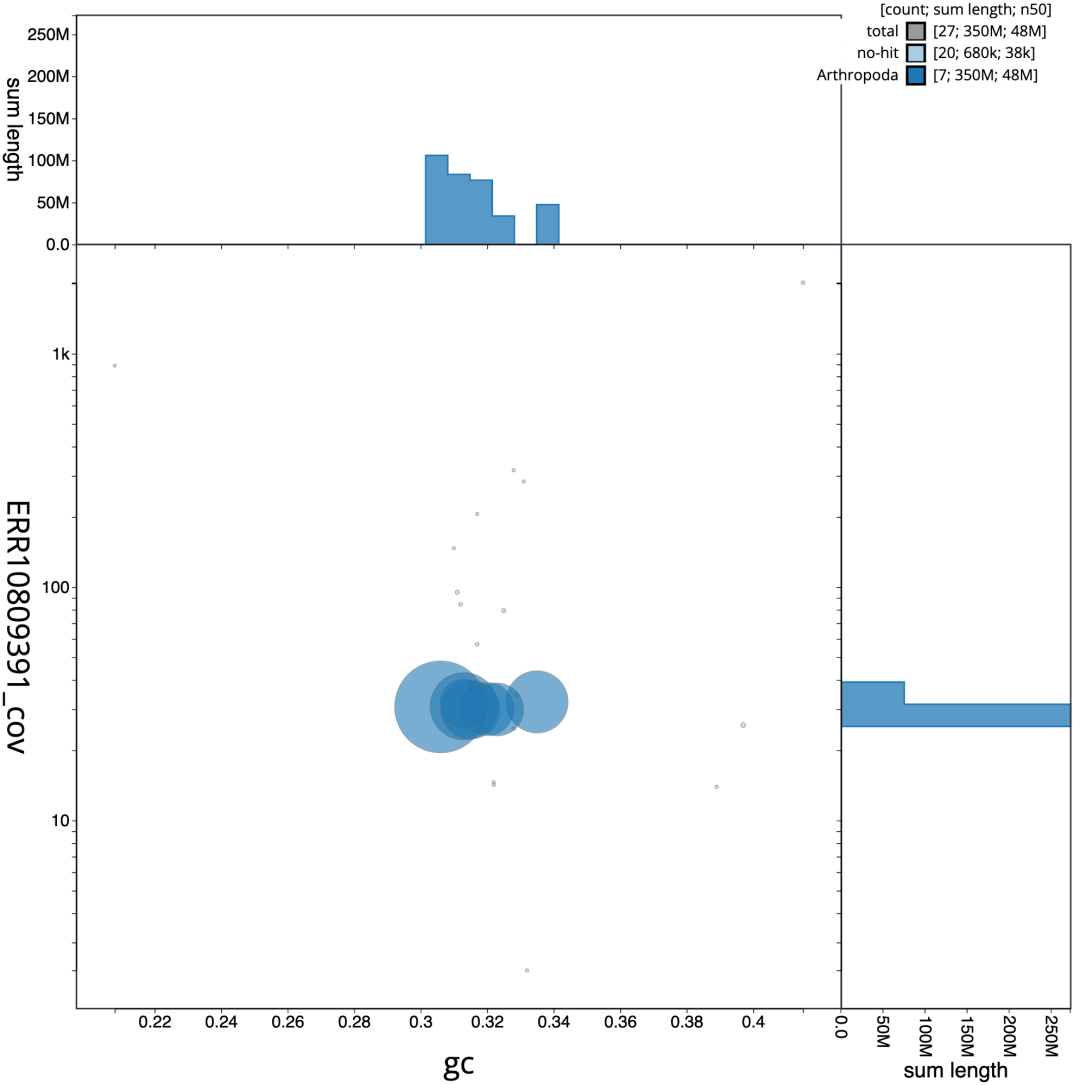
Genome assembly of *Cantharis flavilabris*, icCanFlav1.1: BlobToolKit GC-coverage plot. Scaffolds are coloured by phylum. Circles are sized in proportion to scaffold length. Histograms show the distribution of scaffold length sum along each axis. An interactive version of this figure is available at https://blobtoolkit.genomehubs.org/view/CASCJT01/dataset/CASCJT01/blob.

**Figure 4.**
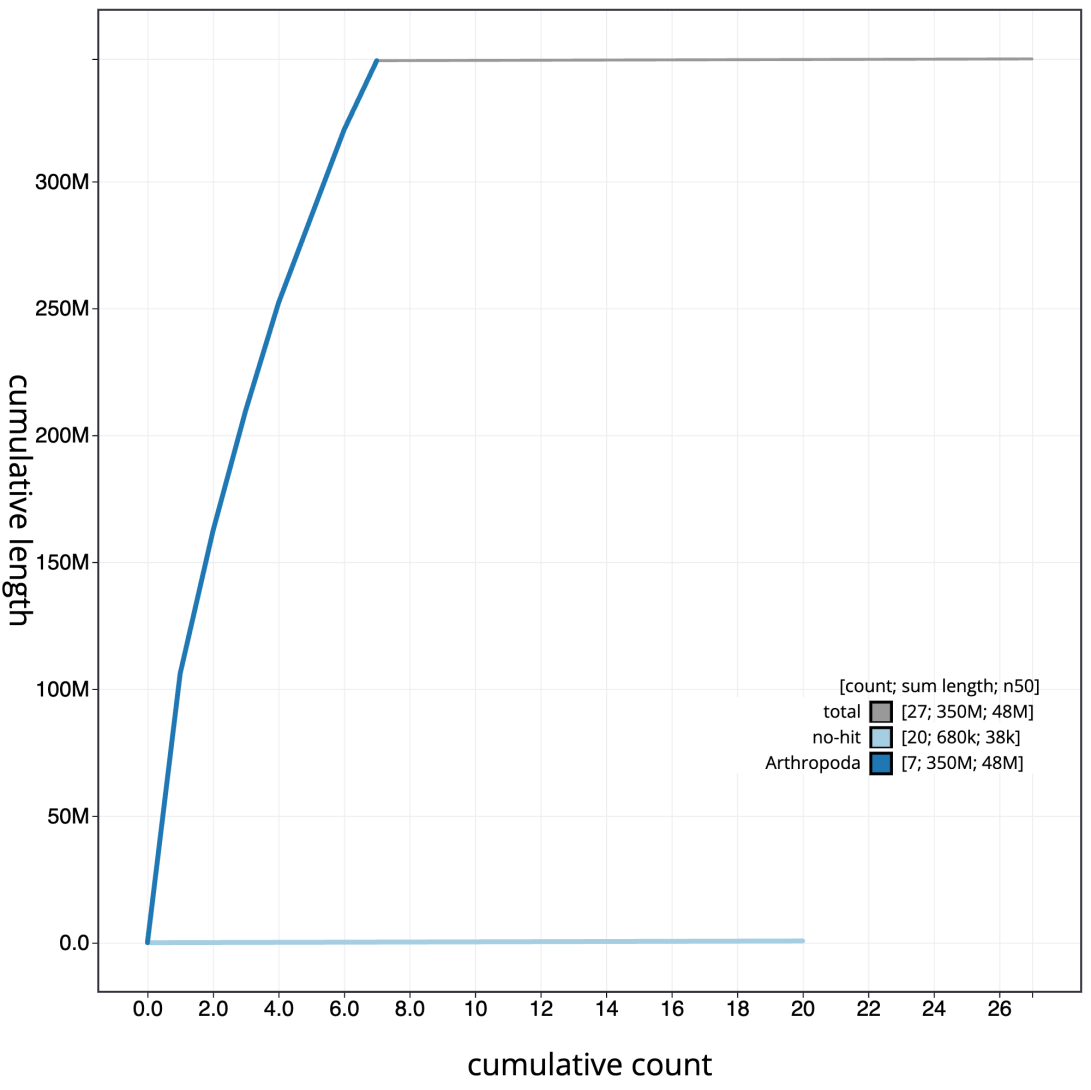
Genome assembly of *Cantharis flavilabris*, icCanFlav1.1: BlobToolKit cumulative sequence plot. The grey line shows cumulative length for all scaffolds. Coloured lines show cumulative lengths of scaffolds assigned to each phylum using the buscogenes taxrule. An interactive version of this figure is available at https://blobtoolkit.genomehubs.org/view/CASCJT01/dataset/CASCJT01/cumulative.

**Figure 5.**
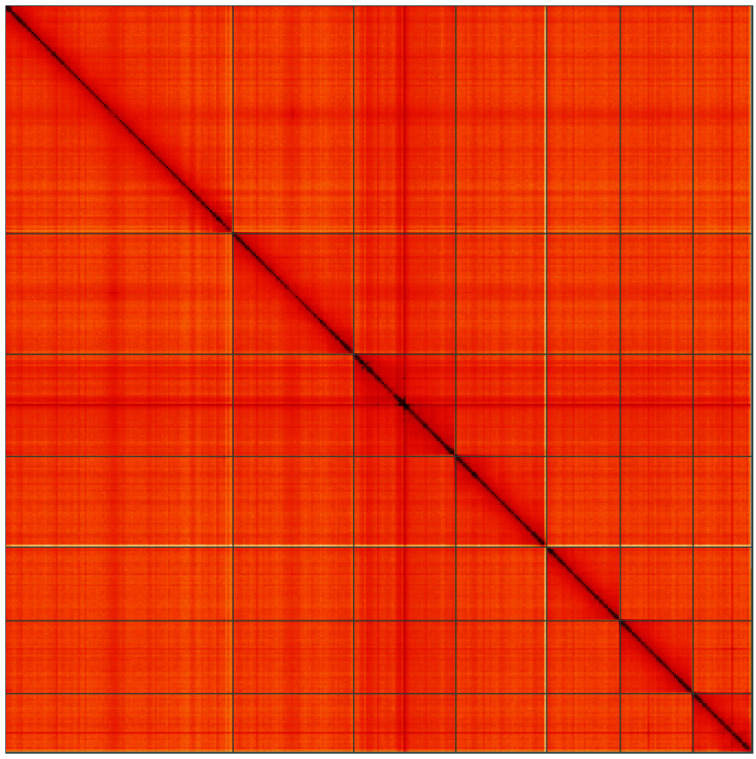
Genome assembly of *Cantharis flavilabris*, icCanFlav1.1: Hi-C contact map of the icCanFlav1.1 assembly, visualised using HiGlass. Chromosomes are shown in order of size from left to right and top to bottom. An interactive version of this figure may be viewed at https://genome-note-higlass.tol.sanger.ac.uk/l/?d=Kkxirt3sTYK9ripEyx-0Yw.

The final assembly has a total length of 348.3 Mb in 26 sequence scaffolds with a scaffold N50 of 47.5 Mb ( Table 1). The snailplot in Figure 2 provides a summary of the assembly statistics, while the distribution of assembly scaffolds on GC proportion and coverage is shown in Figure 3. The cumulative assembly plot in Figure 4 shows curves for subsets of scaffolds assigned to different phyla. Most (99.8%) of the assembly sequence was assigned to 7 chromosomal-level scaffolds, representing 6 autosomes and the X sex chromosome. Chromosome-scale scaffolds confirmed by the Hi-C data are named in order of size ( Figure 5; Table 2). Chromosome X was assigned by synteny to *Cantharis rufa* (GCA_947369205.1) ( Sivell *et al.*, 2023) and *Cantharis rustica* (GCA_911387805.1) ( Sivell *et al.*, 2021). While not fully phased, the assembly deposited is of one haplotype. Contigs corresponding to the second haplotype have also been deposited. The mitochondrial genome was also assembled and can be found as a contig within the multifasta file of the genome submission.

**Table 1.** Genome data for *Cantharis flavilabris*, icCanFlav1.1.

Project accession data		
Assembly identifier	icCanFlav1.1	
Species	*Cantharis flavilabris*	
Specimen	icCanFlav1	
NCBI taxonomy ID	877752	
BioProject	PRJEB59137	
BioSample ID	SAMEA111458455	
Isolate information	icCanFlav1, female: thorax (DNA sequencing), head (Hi-C sequencing)	
Assembly metrics *		*Benchmark*
Consensus quality (QV)	66.2	*≥ 50*
*k*-mer completeness	100.0%	*≥ 95%*
BUSCO **	C:98.7%[S:96.1%,D:2.6%], F:0.6%,M:0.7%,n:2,124	*C ≥ 95%*
Percentage of assembly mapped to chromosomes	99.8%	*≥ 95%*
Sex chromosomes	X	*localised* *homologous pairs*
Organelles	Mitochondrial genome: 17.5 kb	*complete single* *alleles*
Raw data accessions		
PacificBiosciences SEQUEL II	ERR10809391	
Hi-C Illumina	ERR10802468	
Genome assembly		
Assembly accession	GCA_949152465.1	
*Accession of alternate haplotype*	GCA_949152385.1	
Span (Mb)	348.3	
Number of contigs	84	
Contig N50 length (Mb)	10.5	
Number of scaffolds	26	
Scaffold N50 length (Mb)	47.5	
Longest scaffold (Mb)	106.12	
Genome annotation		
Number of protein-coding genes	22,711	
Number of gene transcripts	23,081	

* Assembly metric benchmarks are adapted from column VGP-2020 of “Table 1: Proposed standards and metrics for defining genome assembly quality” from ( Rhie *et al.*, 2021). ** BUSCO scores based on the endopterygota_odb10 BUSCO set using version 5.3.2. C = complete [S = single copy, D = duplicated], F = fragmented, M = missing, n = number of orthologues in comparison. A full set of BUSCO scores is available at https://blobtoolkit.genomehubs.org/view/CASCJT01/dataset/CASCJT01/busco.

**Table 2.** Chromosomal pseudomolecules in the genome assembly of *Cantharis flavilabris*, icCanFlav1.

INSDC accession	Chromosome	Length (Mb)	GC%
OX424435.1	1	106.12	30.5
OX424436.1	2	56.15	31.5
OX424437.1	3	47.55	33.5
OX424438.1	4	42.28	31.5
OX424439.1	5	34.3	32.0
OX424440.1	6	33.9	32.5
OX424441.1	X	27.33	31.5
OX424442.1	MT	0.02	21.0

The estimated Quality Value (QV) of the final assembly is 66.2 with *k*-mer completeness of 100.0%, and the assembly has a BUSCO v5.3.2 completeness of 98.7% (single = 96.1%, duplicated = 2.6%), using the endopterygota_odb10 reference set ( *n* = 2,124).

Metadata for specimens, barcode results, spectra estimates, sequencing runs, contaminants and pre-curation assembly statistics are given at https://links.tol.sanger.ac.uk/species/877752.

## Genome annotation report

The *Cantharis flavilabris* genome assembly (GCA_949152465.1) was annotated using the Ensembl rapid annotation pipeline ( Table 1; https://rapid.ensembl.org/Cantharis_flavilabris_GCA_949152465.1/Info/Index). The resulting annotation includes 23,081 transcribed mRNAs from 22,711 protein-coding genes.

## Methods

### Sample acquisition and nucleic acid extraction

A female *Cantharis flavilabris* (specimen ID NHMUK014439758, ToLID icCanFlav1) was collected from Leith Hill, England, UK (latitude 51.18, longitude –0.37) on 2021-06-20. The specimen was collected and identified by Maxwell Barclay (Natural History Museum) and preserved by dry freezing at –80 °C.

The workflow for high molecular weight (HMW) DNA extraction at the WSI includes a sequence of core procedures: sample preparation; sample homogenisation, DNA extraction, fragmentation, and clean-up. In sample preparation, the icCanFlav1 sample was weighed and dissected on dry ice ( Jay *et al.*, 2023). Tissue from the thorax was homogenised using a PowerMasher II tissue disruptor ( Denton *et al.*, 2023a). HMW DNA was extracted using the Automated MagAttract v1 protocol ( Oatley *et al.*, 2023a). HMW DNA was sheared into an average fragment size of 12–20 kb in a Megaruptor 3 system with speed setting 30 ( Todorovic *et al.*, 2023). Sheared DNA was purified by solid-phase reversible immobilisation ( Oatley *et al.*, 2023b): in brief, the method employs a 1.8X ratio of AMPure PB beads to sample to eliminate shorter fragments and concentrate the DNA. The concentration of the sheared and purified DNA was assessed using a Nanodrop spectrophotometer and Qubit Fluorometer and Qubit dsDNA High Sensitivity Assay kit. Fragment size distribution was evaluated by running the sample on the FemtoPulse system.

Protocols developed by the Wellcome Sanger Institute (WSI) Tree of Life core laboratory have been deposited on protocols.io ( Denton *et al.*, 2023b).

### Sequencing

Pacific Biosciences HiFi circular consensus DNA sequencing libraries were constructed according to the manufacturers’ instructions. DNA sequencing was performed by the Scientific Operations core at the WSI on a Pacific Biosciences SEQUEL II instrument. Hi-C data were also generated from head tissue of icCanFlav1 using the Arima2 kit and sequenced on the Illumina NovaSeq 6000 instrument.

### Genome assembly, curation and evaluation

Assembly was carried out with Hifiasm ( Cheng *et al.*, 2021) and haplotypic duplication was identified and removed with purge_dups ( Guan *et al.*, 2020). The assembly was then scaffolded with Hi-C data ( Rao *et al.*, 2014) using YaHS ( Zhou *et al.*, 2023). The assembly was checked for contamination and corrected as described previously ( Howe *et al.*, 2021). Manual curation was performed using HiGlass ( Kerpedjiev *et al.*, 2018) and PretextView ( Harry, 2022). The mitochondrial genome was assembled using MitoHiFi ( Uliano-Silva *et al.*, 2023), which runs MitoFinder ( Allio *et al.*, 2020) or MITOS ( Bernt *et al.*, 2013) and uses these annotations to select the final mitochondrial contig and to ensure the general quality of the sequence.

A Hi-C map for the final assembly was produced using bwa-mem2 ( Vasimuddin *et al.*, 2019) in the Cooler file format ( Abdennur & Mirny, 2020). To assess the assembly metrics, the *k*-mer completeness and QV consensus quality values were calculated in Merqury ( Rhie *et al.*, 2020). This work was done using Nextflow ( Di Tommaso *et al.*, 2017) DSL2 pipelines “sanger-tol/readmapping” ( Surana *et al.*, 2023a) and “sanger-tol/genomenote” ( Surana *et al.*, 2023b). The genome was analysed within the BlobToolKit environment ( Challis *et al.*, 2020) and BUSCO scores ( Manni *et al.*, 2021; Simão *et al.*, 2015) were calculated.

Table 3 contains a list of relevant software tool versions and sources.

**Table 3.** Software tools: versions and sources.

Software tool	Version	Source
BlobToolKit	4.1.7	https://github.com/blobtoolkit/blobtoolkit
BUSCO	5.3.2	https://gitlab.com/ezlab/busco
Hifiasm	0.16.1-r375	https://github.com/chhylp123/hifiasm
HiGlass	1.11.6	https://github.com/higlass/higlass
Merqury	MerquryFK	https://github.com/thegenemyers/MERQURY.FK
MitoHiFi	2	https://github.com/marcelauliano/MitoHiFi
PretextView	0.2	https://github.com/wtsi-hpag/PretextView
purge_dups	1.2.3	https://github.com/dfguan/purge_dups
sanger-tol/ genomenote	v1.0	https://github.com/sanger-tol/genomenote
sanger-tol/ readmapping	1.1.0	https://github.com/sanger-tol/readmapping/tree/1.1.0
YaHS	1.2a	https://github.com/c-zhou/yahs

### Genome annotation

The BRAKER2 pipeline ( Brůna *et al.*, 2021) was used in the default protein mode to generate annotation for the *Cantharis flavilabris* assembly (GCA_949152465.1) in Ensembl Rapid Release.

### Wellcome Sanger Institute – Legal and Governance

The materials that have contributed to this genome note have been supplied by a Darwin Tree of Life Partner. The submission of materials by a Darwin Tree of Life Partner is subject to the **‘Darwin Tree of Life Project Sampling Code of Practice’**, which can be found in full on the Darwin Tree of Life website here. By agreeing with and signing up to the Sampling Code of Practice, the Darwin Tree of Life Partner agrees they will meet the legal and ethical requirements and standards set out within this document in respect of all samples acquired for, and supplied to, the Darwin Tree of Life Project. 

Further, the Wellcome Sanger Institute employs a process whereby due diligence is carried out proportionate to the nature of the materials themselves, and the circumstances under which they have been/are to be collected and provided for use. The purpose of this is to address and mitigate any potential legal and/or ethical implications of receipt and use of the materials as part of the research project, and to ensure that in doing so we align with best practice wherever possible. The overarching areas of consideration are:

• Ethical review of provenance and sourcing of the material

• Legality of collection, transfer and use (national and international) 

Each transfer of samples is further undertaken according to a Research Collaboration Agreement or Material Transfer Agreement entered into by the Darwin Tree of Life Partner, Genome Research Limited (operating as the Wellcome Sanger Institute), and in some circumstances other Darwin Tree of Life collaborators.

## Data Availability

European Nucleotide Archive: *Cantharis flavilabris*. Accession number PRJEB59137; https://identifiers.org/ena.embl/PRJEB59137 ( Wellcome Sanger Institute, 2023). The genome sequence is released openly for reuse. The *Cantharis flavilabris* genome sequencing initiative is part of the Darwin Tree of Life (DToL) project. All raw sequence data and the assembly have been deposited in INSDC databases. Raw data and assembly accession identifiers are reported in Table 1.

## Author information

Members of the Natural History Museum Genome Acquisition Lab are listed here: https://doi.org/10.5281/zenodo.7139035.

Members of the Darwin Tree of Life Barcoding collective are listed here: https://doi.org/10.5281/zenodo.4893703.

Members of the Wellcome Sanger Institute Tree of Life Management, Samples and Laboratory team are listed here: https://doi.org/10.5281/zenodo.10066175.

Members of Wellcome Sanger Institute Scientific Operations: Sequencing Operations are listed here: https://doi.org/10.5281/zenodo.10043364.

Members of the Wellcome Sanger Institute Tree of Life Core Informatics team are listed here: https://doi.org/10.5281/zenodo.10066637.

Members of the Tree of Life Core Informatics collective are listed here: https://doi.org/10.5281/zenodo.5013541.

Members of the Darwin Tree of Life Consortium are listed here: https://doi.org/10.5281/zenodo.4783558.
