# Supplementary material for: The genome sequence of a soldier beetle, Cantharis flavilabris Fallén, 1807
Source: Wellcome Open Res. 2024 Oct 15;9:303. Originally published 2024 Jun 11. [Version 2] doi: 10.12688/wellcomeopenres.22422.2 (PMC11399767; doi:10.12688/wellcomeopenres.22422.2)
Supplement: Peer review history

# Reviewer report: Reviewer response for version 2

**DOI:** 10.21956/wellcomeopenres.25600.r109310

**Published:** 2024-11-19

**Authors:** Doga Cedden

**Affiliations:** 1 University of Göttingen, Göttingen, Germany

The revised data note by Barclay presents a high-quality genome assembly from the soldier beetle *Cantharis flavilabris. In the revised version, the author provides a detailed explanation of the methods for Hi-C preparation and library preparation and sequencing. As with the other submissions from the Darwin Tree of Life Consortium, the method is appropriate for genome sequencing, and sufficient quality control is provided. The data is publicly available through the provided link. For future submissions, it would be beneficial to add a scale to the photograph of the specimen. I have no further comments.*

Are sufficient details of methods and materials provided to allow replication by others?

Yes

Is the rationale for creating the dataset(s) clearly described?

Yes

Are the datasets clearly presented in a useable and accessible format?

Yes

Are the protocols appropriate and is the work technically sound?

Yes

Reviewer Expertise:

I work on coleopteran pests using RNA-seq and RNAi.

I confirm that I have read this submission and believe that I have an appropriate level of expertise to confirm that it is of an acceptable scientific standard.

# Reviewer report: Reviewer response for version 2

**DOI:** 10.21956/wellcomeopenres.25600.r105081

**Published:** 2024-10-16

**Authors:** Will Nash

**Affiliations:** 1 Research Faculty, Earlham Institute, Norwich Research Park, Norwich, England, UK

Great to see this note updated to have a background section befitting of this attractive beetle. 

 It is also good to see the Hi-C protocol explained in more detail in the methods. 

 These two corrections address my concerns and I am happy to recommend this paper for publication

Are sufficient details of methods and materials provided to allow replication by others?

No

Is the rationale for creating the dataset(s) clearly described?

Yes

Are the datasets clearly presented in a useable and accessible format?

Yes

Are the protocols appropriate and is the work technically sound?

Yes

Reviewer Expertise:

Genome assembly, population genomics, bioinformatics

I confirm that I have read this submission and believe that I have an appropriate level of expertise to confirm that it is of an acceptable scientific standard.

# Reviewer report: Reviewer response for version 1

**DOI:** 10.21956/wellcomeopenres.24701.r90895

**Published:** 2024-09-13

**Authors:** Romain Villoutreix

**Affiliations:** 1 Universite de Montpellier, Montpellier, Occitanie, France

Barclay and colleagues present a genome assembly for the soldier beetle *Cantharis flavilabris.*

 Great care was taken in identifying an individual of the target species and producing this assembly.

 The authors used cutting edge data and pipelines to produce an excellent chromosomal level assembly. This ressource will be of great use to perform genomic work in this species.

 I therefore recommend this article.

Are sufficient details of methods and materials provided to allow replication by others?

Yes

Is the rationale for creating the dataset(s) clearly described?

Yes

Are the datasets clearly presented in a useable and accessible format?

Yes

Are the protocols appropriate and is the work technically sound?

Yes

Reviewer Expertise:

Evolutionary genomics

I confirm that I have read this submission and believe that I have an appropriate level of expertise to confirm that it is of an acceptable scientific standard.

# Reviewer report: Reviewer response for version 1

**DOI:** 10.21956/wellcomeopenres.24701.r87914

**Published:** 2024-08-06

**Authors:** Will Nash

**Affiliations:** 1 Research Faculty, Earlham Institute, Norwich Research Park, Norwich, England, UK

The genome of a soldier beetle is presented. It is sequenced from 74x of PacBioHifi and scaffolded with Hi-C.

 The assembly is 348.3 Mb over 26 scaffolds with an N50 of 47.5Mb. 23,081 genes are annotated. 

 It is dissapointing to see no background included. This does not provide any detail on this species or the biological context of this assembly. 

 The methods are cutting edge in line with other DToL genomes. The Hi-C protocol is not repeatable, and as such I cannot recommend this manuscript.

Are sufficient details of methods and materials provided to allow replication by others?

No

Is the rationale for creating the dataset(s) clearly described?

Yes

Are the datasets clearly presented in a useable and accessible format?

Yes

Are the protocols appropriate and is the work technically sound?

Yes

Reviewer Expertise:

Genome assembly, population genomics, bioinformatics

I confirm that I have read this submission and believe that I have an appropriate level of expertise to state that I do not consider it to be of an acceptable scientific standard, for reasons outlined above.

# Reviewer report: Reviewer response for version 1

**DOI:** 10.21956/wellcomeopenres.24701.r89176

**Published:** 2024-08-06

**Authors:** Yangzi Wang

**Affiliations:** 1 University of Mainz, Mainz, Germany

The manuscript titled "The genome sequence of a soldier beetle, *Cantharis flavilabris* Fallén, 1807" presents a high-quality genome assembly for the soldier beetle, *Cantharis flavilabris*. The authors have employed a combination of robust methodological frameworks that include third-generation long-read sequencing technology, Hi-C, rigorous data curation, and reliably automated gene annotation processes. Below is a detailed evaluation of the manuscript.

 The project demonstrates a high level of professionalism. The genome data has been deposited in NCBI and EMBL, ensuring transparency and easy accessibility. The accessibility of the genome assembly and the raw data has been verified, confirming that it matches the descriptions provided in the manuscript. The authors have utilized a combination of PacBio HiFi long reads and Hi-C data to achieve a high-quality assembly. Manual curation further improved the assembly by correcting missing joins and removing haplotypic duplications. This approach has resulted in a polished genome sequence. The evaluation shows the genome assembly presented is of high quality, with several key measures highlighting its completeness and accuracy: 348.3 Mb genome span; 47.5 Mb Scaffold N50; Chromosome-level assembly; 98.7% BUSCO Completeness. These data suggest that the assembly is complete and highly contiguous, providing a reliable reference genome for further studies. In addition to the nuclear genome, the mitochondrial genome was also assembled, measuring 17.5 kb in length. This presents another valuable genetic dataset for the species. The Ensembl rapid annotation pipeline was employed to annotate the genome, resulting in the identification of 22,711 protein-coding genes. This automated process ensures consistency and accuracy in gene prediction, which paves the way for further functional genomics studies. Furthermore, the manuscript includes detailed figures illustrating the genome assembly statistics, scaffold distribution, and other key aspects.

 There is a minor error in the manuscript: the raw data identifiers ERR10809391 and ERR10802468 appear to be from NCBI SRA, not INSDC, as mentioned. I only found the data using these accession numbers on NCBI. Please correct this in the manuscript. Additionally, I recommend including both accession numbers in the Data Availability section to facilitate quick data access for readers.

 In conclusion, this manuscript represents a valuable genomic dataset of the soldier beetle for the research community. The authors have produced a high-quality genome assembly for the soldier beetle using cutting-edge sequencing technologies and stringent data curation. The availability of this genome assembly in public databases, along with the comprehensive methodological details provided, ensures that this work will be a valuable resource for future research. The authors are to be commended for their professionalism and the high standards of their work.

Are sufficient details of methods and materials provided to allow replication by others?

Yes

Is the rationale for creating the dataset(s) clearly described?

Yes

Are the datasets clearly presented in a useable and accessible format?

Yes

Are the protocols appropriate and is the work technically sound?

Yes

Reviewer Expertise:

Genomics, population genomics, epigenomics

I confirm that I have read this submission and believe that I have an appropriate level of expertise to confirm that it is of an acceptable scientific standard.
